# Development of a Minimal Photosystem for Hydrogen Production in Inorganic Chemical Cells

**DOI:** 10.1002/anie.201805584

**Published:** 2018-09-04

**Authors:** Keita Nakanishi, Geoffrey J. T. Cooper, Laurie J. Points, Leanne G. Bloor, Masaaki Ohba, Leroy Cronin

**Affiliations:** ^1^ WestCHEM School of Chemistry University of Glasgow University Avenue Glasgow G12 8QQ UK; ^2^ Department of Chemistry Faculty of Sciences Kyushu University 744 Motooka Nishi-ku Fukuoka Japan

**Keywords:** chemobrionics, compartmentalisation, hydrogen, iCHELLs, photosynthesis

## Abstract

Inorganic chemical cells (iCHELLs) are compartment structures consisting of polyoxometalates (POMs) and cations, offering structured and confined reaction spaces bounded by membranes. We have constructed a system capable of efficient anisotropic and hierarchical photo‐induced electron transfer across the iCHELL membrane. Mimicking photosynthesis, our system uses proton gradients between the compartment and the bulk to drive efficient conversion of light into chemical energy, producing hydrogen upon irradiation. This illustrates the power of the iCHELL approach for catalysis, where the structure, compartmentalisation and variation in possible components could be utilised to approach a wide range of reactions.

Photosynthesis is the process by which plants and other organisms convert solar energy to chemical energy. It is a widely studied topic due to the many potential applications of synthetic photochemical systems for sustainable solar fuels, such as hydrogen.^1^, ^2^ In living cells, positional assembly is vital for achieving the anisotropic electron, energy, and material transfer required for efficient photosynthesis.^3^, ^4^ Compartmentalisation is also crucial to construct a space which is favourable for specific chemical reactions,^5^ and to partition species; proton gradients across membranes being the energy currency used to drive biochemical reactions. Hydrogen is vital in the modern world economy and has great potential as a clean‐burning fuel. However, currently around 95 % of H_2_ is obtained by reforming fossil fuels, a process that is unsustainable and leads to a net increase in atmospheric CO_2_. The electrolysis of water is a mature, scalable technology for H_2_ production and if the energy source is renewable, for example, sunlight, the process can be sustainable.^6^, ^7^

In order to mimic the compartmentalisation advantages found in cells, self‐assembled soft materials (e.g. liposomes, polymersomes) have often been used as platforms.^8^ However, the building blocks for such materials are often restricted to amphiphilic lipids and high‐molecular weight polymers.^5^, ^8^, ^9^ Catalytic reactions requiring nano‐spaces and cascade reactions have been achieved by designing precisely controlled reaction systems within these platforms.^10^, ^11^, ^12^, ^13^, ^14^, ^15^ Confined spaces can also improve efficiency and selectivity of some catalytic reactions.^16^, ^17^, ^18^ There are numerous examples of photochemical water splitting and artificial photosynthesis,^1^, ^6^ including compartmentalisation of such systems into lipid vesicles.^19^ However, there are no known examples where a chemobrionic system, based on precipitation membranes, has successfully been applied in catalysis, despite the potential advantages of these materials.^20^

Herein, we used inorganic chemical cells (iCHELLs)^21^ as confined reaction spaces in which to compartmentalise the photochemical hydrogen evolution reaction (HER). Careful building block selection allowed us to use proton gradients, providing optimum conditions for efficiency, mimicking the action of photosynthetic cells converting light energy into chemical energy. We used a well‐known four‐component photo‐driven HER system consisting of a photosensitizer ([Ru(bpy)_3_]^2+^, bpy=2,2′‐bipyridyl), an electron mediator (methyl viologen (MV)), a catalyst (Pt colloid), and a sacrificial electron donor (triethanolamine (TEOA)).^22^, ^23^ By constructing iCHELLs composed of [PW_12_O_40_]^3−^ or [SiW_12_O_40_]^4−^ polyoxometalate (POM) anions and MV^2+^ cations, we were able to construct a system in which efficient and sustained HER was driven by anisotropic and hierarchical electron transfer across the membrane, see Figure 1.

**Figure 1:**
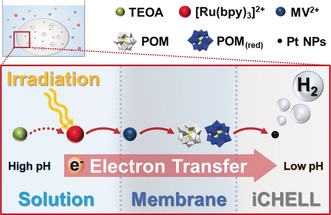
A schematic representation of photo‐driven HER with iCHELLs and multi‐step electron transfer across the membrane. The four component HER system comprises a photosensitizer ([Ru(bpy)_3_]^2+^), electron mediator (methyl viologen (MV)), a catalyst (Pt colloid), and a sacrificial electron donor (triethanolamine (TEOA)). The iCHELL is constructed from POM anions, which also facilitate electron transfer across the membrane.

iCHELLs are hybrid inorganic chemical cells consisting of organic cations and POM anions.^21^ The cell‐like structures are precipitation membranes, fabricated by injection of dense aqueous solution of POM anions (whose countercations are small—H^+^, Na^+^ etc.), into a similarly dense aqueous solution of larger cations (Methylene Blue, [Ru^II^(bpy)_3_]Cl_2_ (bpy=2,2′‐bipyridyl) etc.). A diverse range of cations and POMs with various functions can be used as building blocks, allowing the membrane to be readily functionalised.^24^ Furthermore, building block selection can be used to tune membrane permeability, or to set up a proton or redox gradient across the membrane.^21^ These features make iCHELLs a unique platform for challenging reactions such as artificial photosynthesis, but prior to this work, there have been no reports of iCHELLs performing specific chemical reactions.

While HER is known to proceed efficiently at low pH, due to the reduction potential of protons being more favourable, the performance of sacrificial electron donors drastically declines at low pH due to degradation.^25^, ^26^ Therefore, in homogenous conditions, a compromise of neutral or basic conditions are required for HER. Furthermore, degradation of MV, catalysed by Pt colloids, occurs in presence of hydrogen and limits HER efficiency.^27^ By offering compartmentalised spaces with a different chemical environment compared to the bulk, iCHELLs remove many of the problems associated with HER in homogeneous conditions. Careful choice of building blocks with suitable redox potentials allowed an anisotropic electron transfer system to be developed on, and across, the membrane, taking advantage of the ability to physically partition regions of high and low pH. Also, by designing a multi‐step electron transfer system (e.g. [Ru(bpy)_3_]^2+^→MV^2+^→POM→Pt colloid), it is possible to separate the MV from the Pt colloid, reducing the problem of MV degradation.

Initially, we investigated iCHELLs composed of [SiW_12_O_40_]^4−^ and [Ru(bpy)_3_]^2+^ as a platform for a photochemical HER. [SiW_12_O_40_]^4−^ is a clear choice here, as it is a POM capable of being incorporated into iCHELLs, and its reduced form is known to drive rapid HER in presence of Pt/C.^7^ Whilst iCHELLs were successfully fabricated (see SI) and an observed clear to black colour change indicated reduction of [SiW_12_O_40_]^4−^ on the membrane, electron transfer across membrane could not be confirmed. This was likely because of reverse electron transfer ([SiW_12_O_40_]^5−^→[Ru(bpy)_3_]^3+^) due to the proximity of the [SiW_12_O_40_]^4−^ and [Ru(bpy)_3_]^2+^. To suppress this reverse electron transfer, the distance between these components was increased by addition of an intermediate electron mediator, MV^2+^, which was integrated into the membrane in the place of [Ru(bpy)_3_]^2+^.

A schematic model for the photochemical HER with iCHELLs along with the strategy above is given in Figure 1. Pt nanoparticles (Pt NPs) and POM are confined within the iCHELLs, while [Ru(bpy)_3_]^2+^, MV^2+^, and TEOA are in the bulk solution. The membrane is primarily composed of POMs and MV^2+^, so hierarchical electron transfer from the outside to the inside of the iCHELL was expected. H_3_[PW_12_O_40_] and H_4_[SiW_12_O_40_] are very strong acids, so the pH inside the iCHELL was very low (<1). Conversely, the pH of the bulk solution was maintained at 7.2 using Bis‐Tris buffer (100 mm). This way, the optimum conditions for efficient HER were achieved without the degradation problems found in homogenous systems. Evaluating the reaction system from the viewpoint of its energy diagram (see SI), the reduction potentials [PW_12_O_40_]^5−^/[HPW_12_O_40_]^6−^ or [SiW_12_O_40_]^5−^/[SiW_12_O_40_]^6−^, are lower than that of a proton at pH <7 (in the case of [SiW_12_O_40_]^5−/6−^, pH<3), and these can be further reduced by MV^+⋅^, so theoretically, HER should occur spontaneously in this system.

Electron transfer across the membrane was initially evaluated using a bulk plane membrane composed of [PW_12_O_40_]^3−^ and MV^2+^. As a result of Xe light irradiation to the membrane and solution, the colour of the upper phase immediately turned dark blue due to the generation of MV^+⋅^. After a short lag period, the colour of the lower phase gradually turned to dark blue from the membrane boundary, see Figure 2 and Figure 3). The UV‐vis spectrum of the lower phase showed characteristic broad absorbance (400–900 nm) of reduced species of [PW_12_O_40_]^3−^ corresponding to W^VI/V^ transition (Figure S7), and it is assumed that the colour change of the lower phase was induced by reduction of [PW_12_O_40_]^3−^. It should be noted that reduced species of [PW_12_O_40_]^3−^ were surprisingly stable even in air, as observed when the membrane was left (without irradiation) for 100 h. This is still under investigation, but the membrane may act as barrier for oxygen.

**Figure 2:**
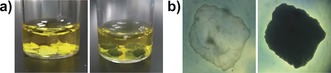
a) Photographs of iCHELLs composed of 100 mm [PW_12_O_40_]^3−^ in 25 mm MV^2+^ before (left), and after (right) 1 h light irradiation. b) Micrographs of one iCHELL (conditions as above) before (left) and after (right) 1 h light irradiation. The iCHELLs shown are approximately 3 mm in diameter.

**Figure 3:**
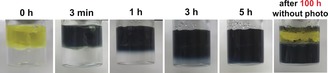
Color changes of the solution during Xe light irradiation from 0–5 h, membrane stability demonstrated by leaving the membrane for 100 h without irradiation. Upper phase; MV, [Ru(bpy)_3_]Cl_2_, TEOA (solvent; Bis‐Tris buffer), Lower phase; H_3_[PW_12_O_40_] (solvent; MilliQ).

As electron transfer across the membrane was confirmed, spherical iCHELLs consisting of MV^2+^ and [PW_12_O_40_]^3−^ were fabricated (see SI for detailed procedure) by injection of a dense aqueous solution of H_3_[PW_12_O_40_] (100 mm) into another dense aqueous solution of MV (25 mm). iCHELLs were formed at various concentrations of H_3_[PW_12_O_40_] (50–500 mm), but those prepared with high concentrations of H_3_[PW_12_O_40_] were found to swell over time due to osmotic pressure, and low concentrations of H_3_[PW_12_O_40_] resulted in flat and fragile iCHELLs. Thus, 100 mm of H_3_[PW_12_O_40_], which resulted in a stable structure, was used for most of the experiments described (6.7 mm over the total reaction volume). As a result of light irradiation, a colour change of iCHELLs to black was observed in presence of [Ru(bpy)_3_]^2+^ and TEOA (Figure 2), indicating that reduction of [PW_12_O_40_]^3−^ also occurred on the iCHELLs.

Photochemical HER was conducted using iCHELLs consisting of POM and MV^2+^ with various POM and Pt NP concentrations (Table 1). Hydrogen gas was successfully produced, and released from the iCHELLS into the reaction headspace, and the highest turnover number (TON) was recorded as TON 3210 (Expt. 3). Although the total volume of hydrogen is small, due to the limited size of the iCHELLs, this TON is higher than that of some similar homogenous systems,^22^, ^23^ although this does not rival the most efficient POM systems.^28^ As a control, iCHELLs composed of MV^2+^ and [PW_12_O_40_]^3−^ broken by ultrasonication were subjected to 24 h light irradiation, and only a trace amount of hydrogen was produced (Table 1, Expt. 8). HER was also conducted without POMs, and again no hydrogen production occurred (Expt. 9). Exclusion of the Pt NP catalyst resulted in a small amount of hydrogen production (Expt. 4), but the presence of platinum accelerated the rate of hydrogen evolution significantly. These results indicated that both the POM and the compartmentalisation within the iCHELL structure were essential to generate hydrogen at this pH range. Photochemical HER with iCHELLs composed of [SiW_12_O_40_]^4−^ and MV^2+^ was also conducted (Expt. 7), but the TON was lower than with [PW_12_O_40_]^3−^. A mixture of H_4_[SiW_12_O_40_] and Pt NPs resulted in small precipitations within a few hours, presumably resulting in low efficiency for the HER.

**Table 1:** Comparison with efficiency of photochemical HER with iCHELLs under various conditions. The reaction was conducted in presence of [Ru(bpy)_3_]Cl_2_ (0.1 mm), MV (25 mm), and TEOA (50 mm). Solvent of outside of iCHELLs was Bis‐Tris buffer (100 mm, pH 7.2). POM and Pt NP concentrations are given with respect to the whole system.

Expt.	H_3_[PW_12_O_40_] [mmol L^−1^]	Pt NPs [μmol L^−1^]	H_2_ produced [μL (μmol)]	TON (vs. Pt)	Reaction time [h]
1	6.7	4.3	100.6 (4.49)	701	12
2	6.7	2.6	100.5 (4.48)	1167	12
3	6.7	0.9	92.1 (4.11)	3210	14
4	6.7	0	10.1 (0.45)	N/A	24
5	5.3	4.3	82.0 (3.66)	571	10
6	8.0	4.3	118.1 (5.27)	823	15
7	6.7 (H_4_[SiW_12_O_40_])	4.3	36.4 (1.63)	254	8
8	6.7	8.5	0.38 (0.02)	1	24
9	0	8.5	0	0	6

Time course measurements for hydrogen production in Experiments 1–4 are shown in Figure 4 a. In each case the plot is divided into three phases; at the beginning of light irradiation, there is an induction period (ca. 0–3 hrs) where only a small amount of hydrogen is produced and the rate of HER is very slow. While in homogenous conditions HER would be expected to begin immediately upon irradiation, HER within iCHELLs is expected to initiate more slowly due to the need to transfer electrons across the membrane. Generation of [HPW_12_O_40_]^6−^ is essential to drive proton reduction, and therefore all the [PW_12_O_40_]^3−^ in the iCHELLs must be reduced to [PW_12_O_40_]^5−^ before hydrogen evolution can begin. After a few hours of irradiation of the iCHELLs, a rapid increase in the HER rate was observed (ca. 3–12 hrs). We hypothesise that in this phase electrons transferred into the inside of the iCHELLs are used for proton reduction directly. After this period, a decay in the hydrogen evolution was observed due to deactivation of [PW_12_O_40_]^3−^ or Pt NPs, or a decreased concentration of protons in the iCHELLs.

**Figure 4:**
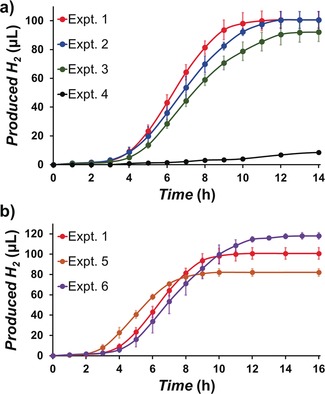
Time‐courses of volume of hydrogen produced during photo‐reaction a) at various Pt concentrations [Expt. 1=4.3, Expt. 2=2.6, Expt. 3=0.9 & Expt. 4=0 μm wrt total solution volume], and b) at various H_3_[PW_12_O_40_] concentrations [Expt. 5=5.3, Expt. 1=6.7 & Expt. 6=8.0 mm wrt total solution volume].

To verify the reason for the decay, photochemical HER was conducted with various initial concentrations of Pt. The total volume of hydrogen produced was similar when the Pt concentration was halved (Expts. 1 & 2), suggesting that the dominant reason for the decay was not deactivation of Pt NPs. However, upon reducing the Pt concentration further (by a factor of ca. 5×), the total volume of hydrogen produced somewhat smaller (Expt. 3), so the effect of Pt availability on the decay is not negligible.

As described previously, H_3_[PW_12_O_40_] is a strong acid, and three protons are almost completely dissociated in aqueous solution. Therefore, increasing the concentration of H_3_[PW_12_O_40_] provides an increased concentration of protons inside of iCHELLs. Time courses of hydrogen production where the concentration of H_3_[PW_12_O_40_] was varied (Expt. 1, 5 & 6) are shown in Figure 4 b. Higher concentration of H_3_[PW_12_O_40_] resulted in a greater volume of hydrogen in total, confirming that the available concentration of protons is strongly linked to the decay of hydrogen production, and defines the total volume of hydrogen that can be produced. The length of induction period also shows a correlation with the H_3_[PW_12_O_40_] concentration. At lower concentrations, the total amount of [PW_12_O_40_]^3−^ in the iCHELLs is small, requiring fewer electrons for reduction to [PW_12_O_40_]^5−^, resulting in a shorter induction period for HER. It should be noted however, that the concentration of H_3_[PW_12_O_40_] also affects morphology of the membrane (shape, thickness etc.), as described above, and these morphological changes could also influence the induction period.

In conclusion, we achieved a minimal photosystem involving hierarchical and anisotropic electron transfer from the photosensitizer across a membrane, and successfully observed photochemical HER from Pt NP inside the iCHELL structure with a high TON. The unique properties of the iCHELLs allowed optimum conditions for both proton reduction and sacrificial electron donor stability by utilising a membrane and providing a compartmentalised low pH environment for HER and a high pH for electron donor stability. An induction period, which is not normally observed in homogeneous HER, was observed in the iCHELLs due to membrane transport, and its length was controllable by changing building block concentration. The effect of size was not investigated here, but it is presumed that larger numbers of smaller iCHELLs would be favourable for HER, due to the larger overall surface area for surface reduction of POMs. Mass‐production and precise size control of iCHELLs by using microfluidic devices,^20^ would allow further optimization to more efficient HER, as would the testing of further combinations of POMs, sacrificial electron donors and electron mediators and photosensitizers. Furthermore, this could allow us to take advantage of the high efficiency and promote iCHELLs as a viable platform for hydrogen production on a useful scale. Having proved the potential of iCHELLs as unique platforms enabling difficult chemical reactions, further applications in water splitting and CO_2_ reduction are under investigation. iCHELLs represent a unique approach to structuring multicomponent catalytic systems in a similar manner to the spatial organisation critical to many biological processes, and one could foresee the ability to design structures with active sites akin to those in enzymes.

## Experimental Section

General experimental remarks: All chemicals were purchased from commercial sources and used without further purification, except for Tris(2,2′‐bipyridyl)ruthenium(II**)** dichloride hexahydrate ([Ru(bpy)_3_]Cl_2_⋅6 H_2_O). Phosphotungstic acid hydrate (H_3_[PW_12_O_40_]⋅*n* H_2_O) was purchased from Wako. Methyl viologen dichloride (MV) and Triethanolamine (TEOA) were purchased from TCI. PVP‐protected colloidal Pt (2 nm in particle size) was purchased from Tanaka Holdings Co., Ltd. Silicotungstic acid hydrate (H_4_[SiW_12_O_40_]⋅*n* H_2_O) and all other materials were purchased from Sigma Aldrich. ^1^H‐NMR analyses were measured with JEOL 600 MHz NMR. UV/Vis absorption spectra were measured with JASCO V‐630. Visible‐light irradiation (400<*λ*<800 nm, 300 W) was conducted using Xenon Light Source XFL‐300.

Fabrication of iCHELLs consisting of [SiW_12_O_40_]^4−^ or [PW_12_O_40_]^3−^ and [Ru(bpy)_3_]^2+^: 20 μL of aqueous solution of POM (H_4_[SiW_12_O_40_] or H_3_[PW_12_O_40_]) (100 mm) was intermittently injected into 1.4 mL of another aqueous buffer solution of [Ru(bpy)_3_]Cl_2_ (26.5 mm) and TEOA (53.5 mm) with micro syringe to form iCHELLs. Volume per one iCHELL was about 5 μL, so approximately four iCHELLs were prepared.

Fabrication of bulk membrane composed of [PW_12_O_40_]^3−^ and MV^2+^: 2.0 mL of aqueous solution of H_3_[PW_12_O_40_] (100 mm) in 10 mL glass vial was degassed by argon bubbling for 30 minutes. 2.0 mL of dichloromethane (DCM) degassed with Ar in advance was added on the top of the H_3_[PW_12_O_40_] solution. Then, 2.0 mL of degassed mixture of MV (25 mm), [Ru(bpy)_3_]Cl_2_⋅6 H_2_O (0.1 mm), and TEOA (50 mm) was also added on the top of dichloromethane. Dichloromethane was removed with syringe, and plane bulk membrane was formed.

Fabrication of iCHELLs consisting of MV and POM for HER: 100 μL of aqueous solution of POM (H_4_[SiW_12_O_40_] or H_3_[PW_12_O_40_]) (100 mm) was intermittently injected into 1.4 mL of another aqueous buffer solution of MV (26.5 mm), [Ru(bpy)_3_]Cl_2_⋅6 H_2_O (0.107 mm), and TEOA (53.5 mm) with micro syringe to form iCHELLs (Scheme S4 in SI). The volume per iCHELL was approximately 5 μL, so around twenty iCHELLs were prepared.

## Conflict of interest

The authors declare no conflict of interest.

## Supporting information

As a service to our authors and readers, this journal provides supporting information supplied by the authors. Such materials are peer reviewed and may be re‐organized for online delivery, but are not copy‐edited or typeset. Technical support issues arising from supporting information (other than missing files) should be addressed to the authors.

Supplementary
