# Supplementary material for: Development of a Minimal Photosystem for Hydrogen Production in Inorganic Chemical Cells
Source: Angew Chem Int Ed Engl. 2018 Sep 4;57(40):13066–70. doi: 10.1002/anie.201805584 (PMC6348376; doi:10.1002/anie.201805584)
Supplement: Supplementary file 1 — Supplementary [file ANIE-57-13066-s001.pdf]

Angewandte Chemie
Eine Zeitschrift der Gesellschaft Deutscher Chemiker

Supporting Information

Development of a Minimal Photosystem for Hydrogen Production in Inorganic Chemical Cells

Keita Nakanishi, Geoffrey J. T. Cooper, Laurie J. Points, Leanne G. Bloor, Masaaki Ohba, and Leroy Cronin**

anie_201805584_sm_miscellaneous_information.pdf

Table of Contents

General Experimental Remarks	S2
Synthesis of $[Ru(bpy)_3]Cl_2 \cdot 6H_2O$	S2
Schematic of photo-driven HER in iCHELLs	S2
Cyclic Voltammetry and Energy Diagrams	S3
Fabrication of iCHELLs and Bulk Membranes	S7
Headspace Hydrogen Determination Data	S9
References	S15

General Experimental Remarks: All chemicals were purchased from commercial sources and used without further purification, except Tris (2,2'-bipyridyl) ruthenium (II) dichloride hexahydrate ($[Ru(bpy)_3]Cl_2{\cdot}6H_2O$). Phosphotungstic acid hydrate ($H_3[PW_{12}O_{40}]{\cdot}nH_2O$) was purchased from Wako. Silicotungstic acid hydrate ($H_4[SiW_{12}O_{40}]{\cdot}nH_2O$) was purchased from Sigma Aldrich. Methyl viologen dichloride (MV) and Triethanolamine (TEOA) were purchased from TCI. PVP-protected colloidal Pt (2 nm in particle size) was purchased from Tanaka Holdings Co.,Ltd. ^{1}H-NMR analyses were measured with JEOL 600MHz NMR. UV-Vis absorption spectra were measured with JASCO V-630. Visible-light irradiation ($400 < \lambda < 800$ nm, 300 W) was conducted using Xenon Light Source XFL-300.

Synthesis of $[Ru(bpy)_3]Cl_2{\cdot}6H_2O$: 3.15g of 2,2'-bipyridine (20 mmol) and 1.00 g of $RuCl_3{\cdot}nH_2O$ (4.0 mmol) were dissolved in 200 mL of ethanol. The mixture was heated at 85˚C with stirring for 72 h, resulting in red solution. The solution was concentrated to *ca.* 5 mL by rotary evaporation after filtration. 200 mL of acetone was added to the solution, and orange powder was precipitated. The orange powder was collected by filtration, and redissolved in minimum volume of hot water. Formation of red crystals immediately began after cooling to room temperature, and the solution was kept at 4˚C for 24h (2.20 g, 2.94 mmol, yield: 77%).

^{1}H-NMR (600 MHz, $CDCl_3$): δ = 7.36 – 7.38 (t, 6H, 2-pyridine), 7.83 – 7.84 (d, 6H, 2-pyridine), 8.04 – 8.06 (t, 6H, 2-pyridine), 8.53 – 8.55 (d, 6H, 2-pyridine)

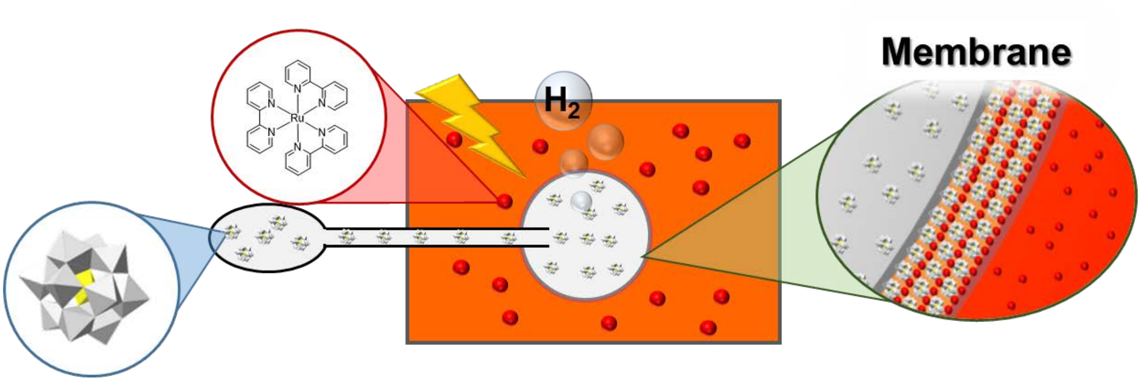

Figure S1. A schematic procedure to fabricate iCHELLs

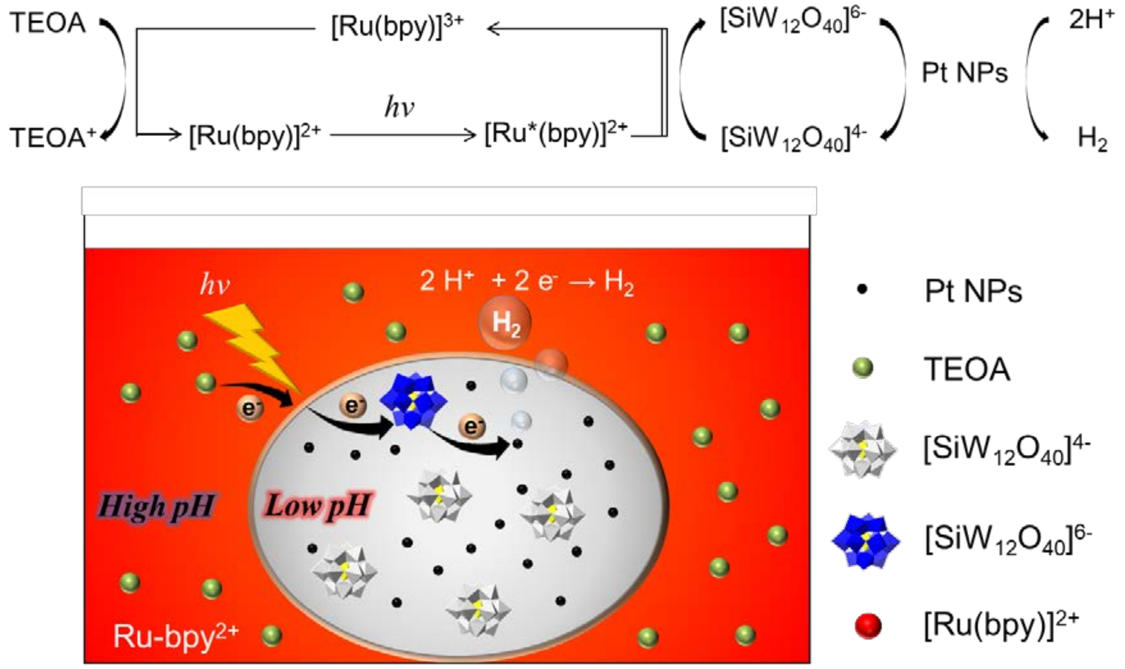

Figure S2. A schematic representation of photo-driven HER with iCHELLs composed of $[Ru(bpy)_3]^{2+}$ and POMs

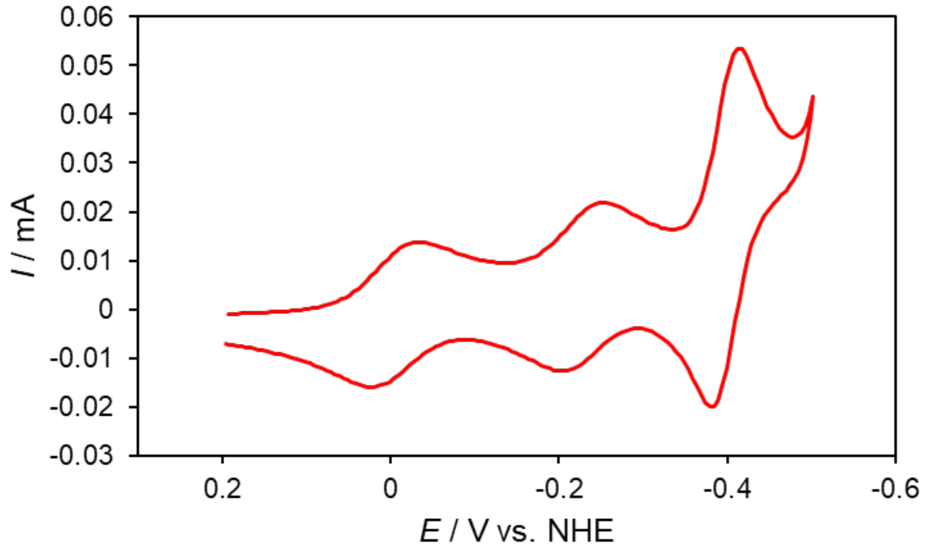

Figure S3. Cyclic voltammogram of $H_4[SiW_{12}O_{40}]$(1.0 mM) in H_2SO_4 (0.50 M) using glassy carbon as a working electrode with scan rate of 100 mV·s $^{-1}$. Three reversible redox waves centered at -0.01 V, -0.23 V and -0.39 V (all potentials are versus the normal hydrogen electrode (NHE)) were observed.

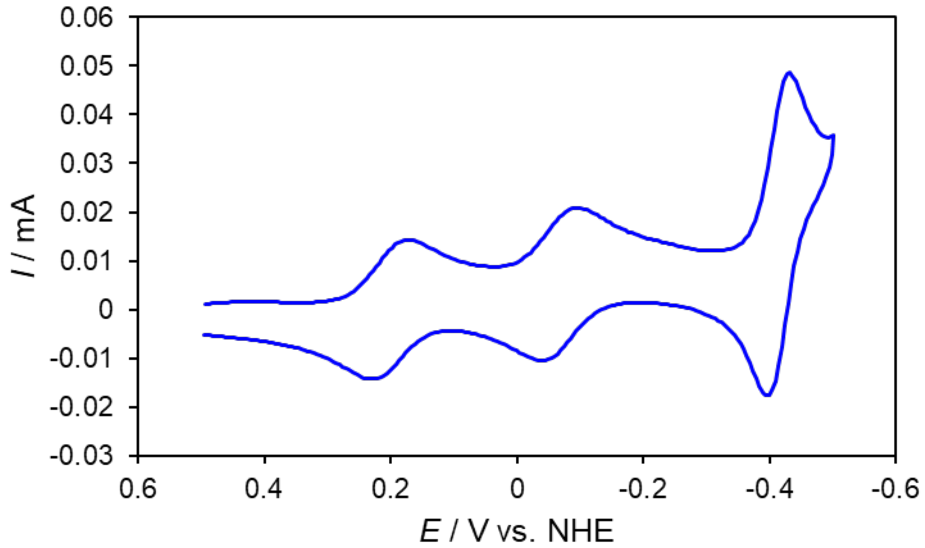

Figure S4. Cyclic voltammogram of $H_3[PW_{12}O_{40}]$(1.0 mM) in H_2SO_4 (0.50 M) using glassy carbon as a working electrode with scan rate of 100 mV·s $^{-1}$. Three reversible redox waves centered at +0.19 V, –0.07 V and -0.42 V (vs.NHE) were observed.

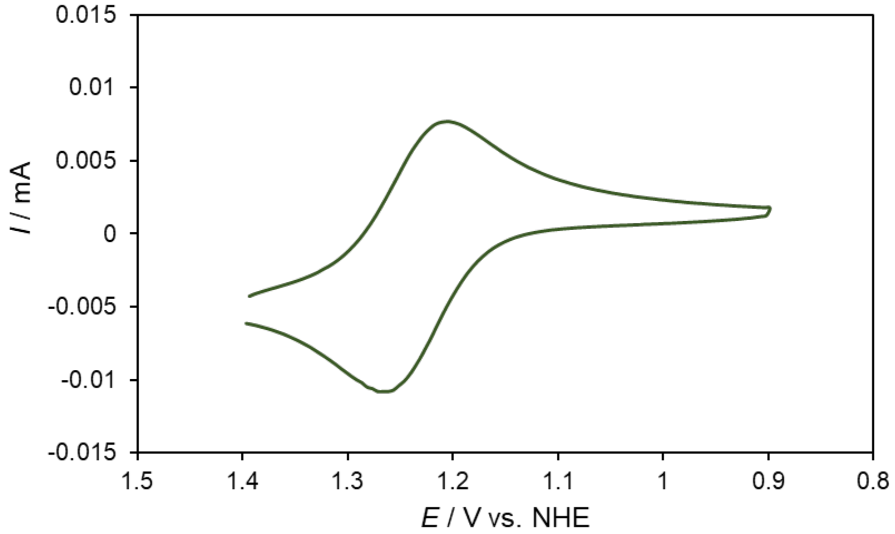

Figure S5. Cyclic voltammogram of $[Ru(bpy)_3]Cl_2$ (1.0 mM) in H_2SO_4 (0.10 M) and Na_2SO_4 (0.9 M) using glassy carbon as a working electrode with scan rate of 100 mV·s $^{-1}$. Only one reversible redox wave centered at 1.24 V (vs.NHE) was observed.

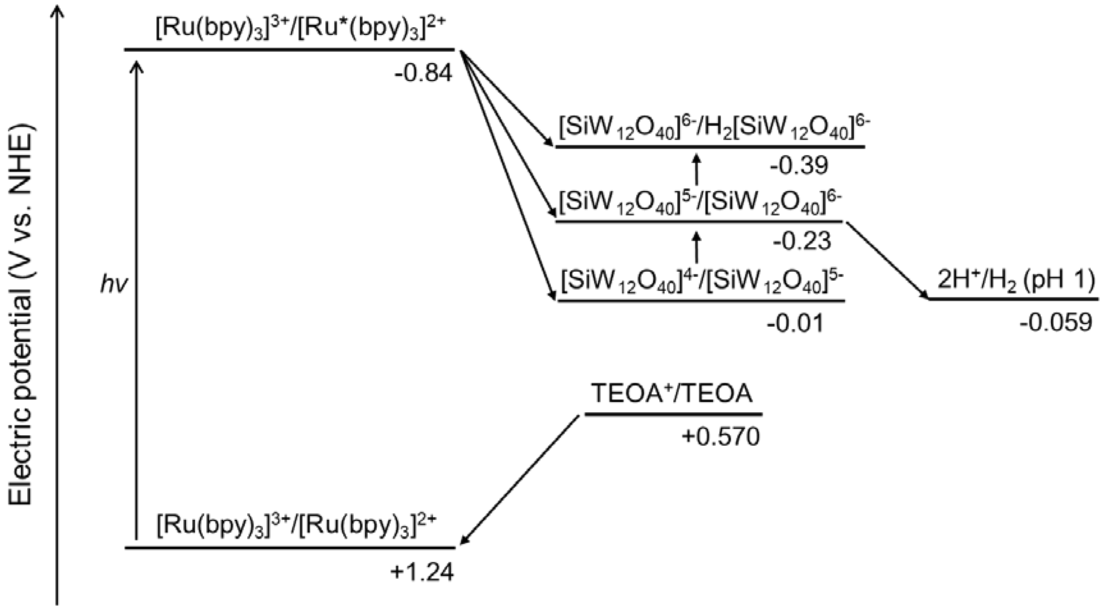

Scheme S1. Energy diagram of the reaction system shown in **Figure S2.** at pH 1

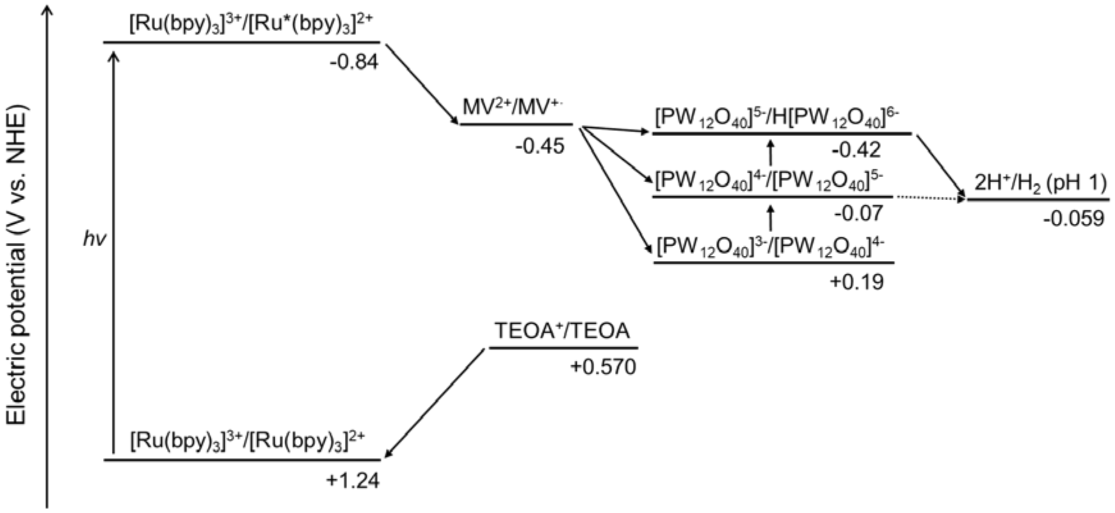

Scheme S2. Energy diagram of multi-step electron transfer and HER with iCHELLs consisting of $[PW_{12}O_{40}]^{3-}$ and MV at pH 1. Redox potential of $MV^{2+}/MV^{+\cdot}$ and $TEOA^{+}/TEOA$ was referred from the literatures.[1,2]

Fabrication of iCHELLs Consisting of $[SiW_{12}O_{40}]^{4-}$ and $[Ru(bpy)_3]^{2+}$: 20 μL of aqueous solution of $H_4[SiW_{12}O_{40}]$ (100 mM) was intermittently injected into 1.4 mL of another aqueous buffer solution of $[Ru(bpy)_3]Cl_2$ (26.5 mM) and TEOA (53.5 mM) with micro syringe to form iCHELLs. Volume per one iCHELL was about 5 μL, so approximately four iCHELLs were prepared.

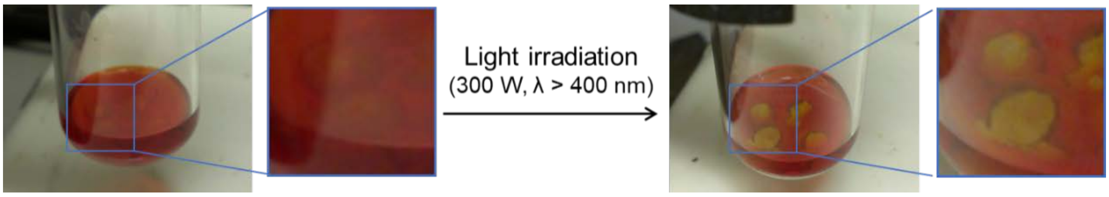

Figure S6. Color change of iCHELLs composed of $[SiW_{12}O_{40}]^{4-}$ and $[Ru(bpy)_3]^{2+}$ associated with photo-irradiation

Fabrication of bulk membrane composed of $[PW_{12}O_{40}]^{3-}$ and MV^{2+}: 2.0 mL of aqueous solution of $H_3[PW_{12}O_{40}]$ (100 mM) in 10 mL glass vial was degassed by argon bubbling for 30 minutes. 2.0 mL of dichloromethane (DCM) degassed with Ar in advance was added on the top of the $H_3[PW_{12}O_{40}]$ solution. Then, 2.0 mL of degassed mixture of MV (25 mM), $[Ru(bpy)_3]Cl_2{\cdot}6H_2O$ (0.1 mM), and TEOA (50 mM) was also added on the top of dichloromethane. Dichloromethane was removed with syringe, and plane bulk membrane was formed (**Scheme S3**).

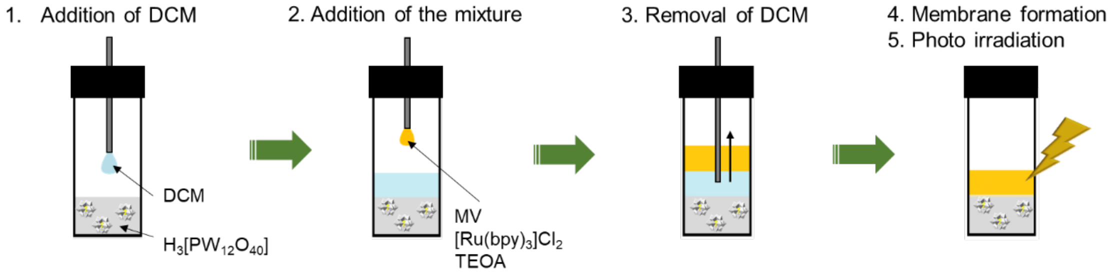

Scheme S3. Procedure to fabricate bulk plane membrane composed of $[PW_{12}O_{40}]^{3-}$ and MV^{2+}

Evaluation of Electron Transfer across Bulk Membrane: Bulk membrane and solution in the glass vial were irradiated with Xe lamp for 10 hours at room temperature. Solution in lower phase was sucked up with syringe, and diluted 100 times to prepare 2.5 mL of aqueous solution of $[PW_{12}O_{40}]^{n-}$ (1.0 mM) in standard cell with screw cap. UV-vis spectrum of this solution was measured under argon atmosphere (**Figure S7**).

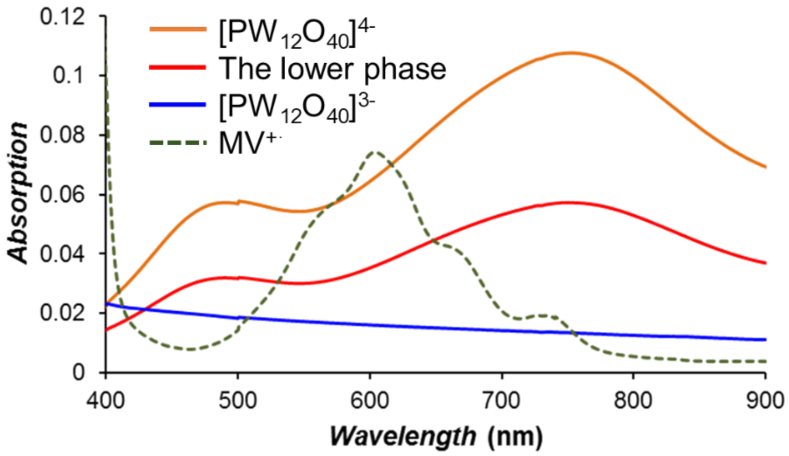

Figure S7. UV-vis spectra of the lower phase solution and $[PW_{12}O_{40}]^{3-}$, $[PW_{12}O_{40}]^{4-}$, and $MV^{+\cdot}$. Reduced species of $[PW_{12}O_{40}]^{3-}$ and $MV^{+\cdot}$ were prepared by bulk electrolysis.

Fabrication of iCHELLs Consisting of MV and Polyoxometalate for HER: 100 μL of aqueous solution of POMs (100 mM) was intermittently injected into 1.4 mL of another aqueous buffer solution of MV (26.5 mM), Ru-bpy (0.107 mM), and TEOA (53.5 mM) with micro syringe to form iCHELLs (**Scheme S4**). Volume per one iCHELL was about 5 μL, so approximately twenty iCHELLs were prepared.

*Tiny amount of Ru-bpy might be integrated into the membrane, but its amount should be very small. Therefore, it is postulated that the membrane was almost completely composed of MV and POMs.

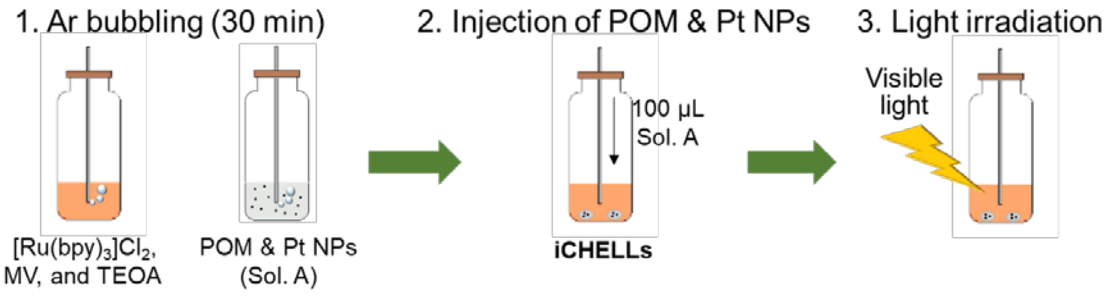

Scheme S4. Procedure to fabricate iCHELLs composed of POMs and MV^{2+} for HER

Photo-chemical Hydrogen Evolution Reaction with iCHELLs: iCHELLs prepared according to the procedure described above were irradiated with Xe lamp at room temperature.

Headspace Hydrogen Determination: Gas chromatography was conducted using GC-8A gas chromatograph equipped with a molecular sieve 5 Å column (3.0 m x 3.0 mm i.d.;30˚C) with Ar carrier gas (200 $mLmin^{-1}$). Headspace gas of the reaction vial was sampled by gas-tight syringe (volume taken per sampling event = 100 μL) and introduced onto GC column by direct injection every 1 hour. The GC oven temperature was set to 60 ºC and the carrier gas was Ar. The front inlet was set to 90 °C. The GC system was calibrated for H_2 using standards gas of pure H_2 (99.99%) supplied by GL Sciences (Japan). Linear fits of volume % vs. peak area were obtained, which allowed peak areas to be converted into volume % of H_2 in the vial headspace. Total system headspaces were calculated by filling the cells with water at room temperature. Typical headspaces were on the order of 11 mL.

Table S1. Time-courses of volume and mole of hydrogen produced during photo-reaction and the average values (Table 1, Experiment 1).

Time (h)	Entry 1		Entry 2		Entry 3		Average		
	vol (μL)	μmol	vol (μL)	μmol	vol (μL)	μmol	vol (μL)	μmol	standard deviation
0	0.00	0.00	0.00	0.00	0.00	0.00	0.00	0.00	0.00
1	0.48	0.02	0.54	0.02	1.42	0.06	0.81	0.04	0.53
2	0.79	0.04	0.71	0.03	2.73	0.12	1.41	0.06	1.15
3	2.02	0.09	1.61	0.07	4.53	0.20	2.72	0.12	1.58
4	6.94	0.31	8.72	0.39	11.72	0.52	9.13	0.41	2.42
5	19.27	0.86	23.24	1.04	27.56	1.23	23.36	1.04	4.15
6	37.01	1.65	46.54	2.08	45.93	2.05	43.16	1.93	5.34
7	58.67	2.62	69.04	3.08	65.02	2.90	64.24	2.87	5.23
8	77.08	3.44	86.17	3.85	80.92	3.61	81.39	3.63	4.56
9	86.76	3.87	100.36	4.48	93.83	4.19	93.65	4.18	6.80
10	89.79	4.01	103.90	4.64	100.75	4.50	98.15	4.38	7.41
11	92.29	4.12	104.85	4.68	102.77	4.59	99.97	4.46	6.73
12	94.21	4.21	104.85	4.68	102.87	4.59	100.64	4.49	5.66

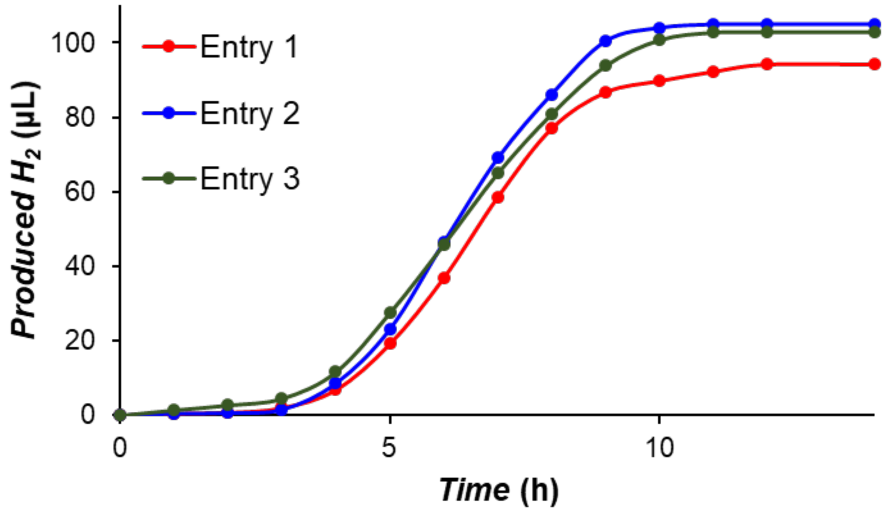

Figure S8. Time-courses of volume of hydrogen produced during photo-reaction (Table 1, Experiment 1).

Table S2. Time-courses of volume and mole of hydrogen produced during photo-reaction and the average values (Table 1, Experiment 2).

Time (h)	Entry 1		Entry 2		Entry 3		Average		
	vol (μL)	μmol	vol (μL)	μmol	vol (μL)	μmol	vol (μL)	μmol	standard deviation
0	0.00	0.00	0.00	0.00	0.00	0.00	0.00	0.00	0.00
1	0.95	0.04	0.76	0.03	1.29	0.06	1.00	0.04	0.27
2	1.40	0.06	1.07	0.05	2.34	0.10	1.60	0.07	0.66
3	2.65	0.12	2.08	0.09	4.79	0.21	3.17	0.14	1.43
4	6.49	0.29	7.45	0.33	12.47	0.56	8.81	0.39	3.21
5	12.82	0.57	21.01	0.94	25.60	1.14	19.81	0.88	6.48
6	26.67	1.19	38.17	1.70	43.11	1.92	35.98	1.61	8.43
7	44.52	1.99	56.93	2.54	59.13	2.64	53.53	2.39	7.88
8	63.52	2.84	71.58	3.20	74.46	3.32	69.86	3.12	5.67
9	79.53	3.55	83.60	3.73	84.23	3.76	82.45	3.68	2.55
10	95.06	4.24	91.09	4.07	90.65	4.05	92.27	4.12	2.43
11	100.82	4.50	95.31	4.25	96.54	4.31	97.56	4.36	2.89
12	107.17	4.78	97.84	4.37	96.54	4.31	100.52	4.49	5.80
13	107.17	4.78	97.88	4.37	96.54	4.31	100.53	4.49	5.79
14	107.17	4.78	97.88	4.37	96.54	4.31	100.53	4.49	5.79

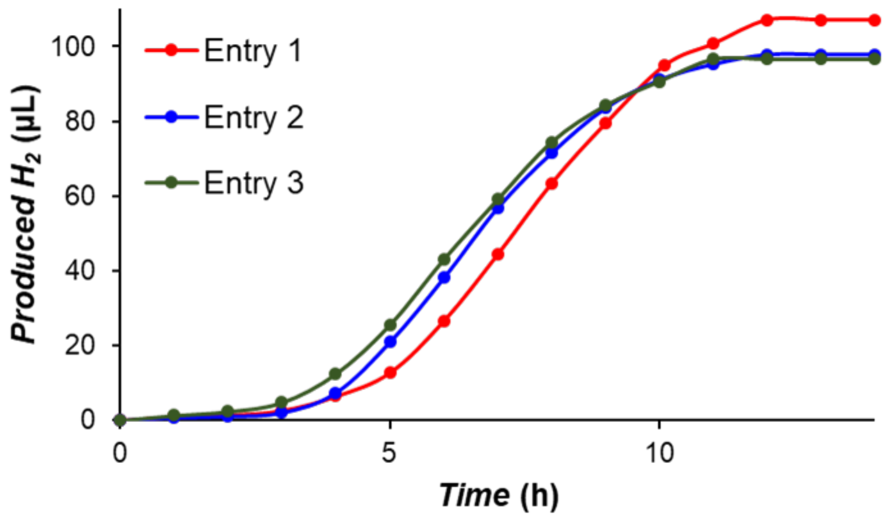

Figure S9. Time-courses of volume of hydrogen produced during photo-reaction (Table 1, Experiment 2).

Table S3. Time-courses of volume and mole of hydrogen produced during photo-reaction and the average values (Table 1, Experiment 3).

Time (h)	Entry 1		Entry 2		Entry 3		Average		
	vol (μL)	μmol	vol (μL)	μmol	vol (μL)	μmol	vol (μL)	μmol	standard deviation
0	0.00	0.00	0.00	0.00	0.00	0.00	0.00	0.00	0.00
1	0.77	0.03	0.80	0.04	0.93	0.04	0.83	0.04	0.09
2	1.12	0.05	1.22	0.05	1.33	0.06	1.22	0.05	0.10
3	1.94	0.09	2.14	0.10	1.67	0.07	1.91	0.09	0.23
4	5.99	0.27	5.31	0.24	4.05	0.18	5.12	0.23	0.99
5	15.46	0.69	14.22	0.63	11.32	0.51	13.67	0.61	2.13
6	31.73	1.42	28.18	1.26	25.53	1.14	28.48	1.27	3.11
7	48.55	2.17	44.00	1.96	40.71	1.82	44.42	1.98	3.94
8	62.97	2.81	58.99	2.63	55.14	2.46	59.03	2.64	3.92
9	75.40	3.37	70.45	3.14	66.18	2.95	70.68	3.16	4.61
10	85.11	3.80	78.79	3.52	72.44	3.23	78.78	3.52	6.33
11	91.45	4.08	86.09	3.84	78.72	3.51	85.42	3.81	6.39
12	97.53	4.35	90.83	4.05	83.21	3.71	90.52	4.04	7.17
13	98.11	4.38	92.94	4.15	84.76	3.78	91.94	4.10	6.73
14	98.11	4.38	93.04	4.15	85.29	3.81	92.15	4.11	6.46

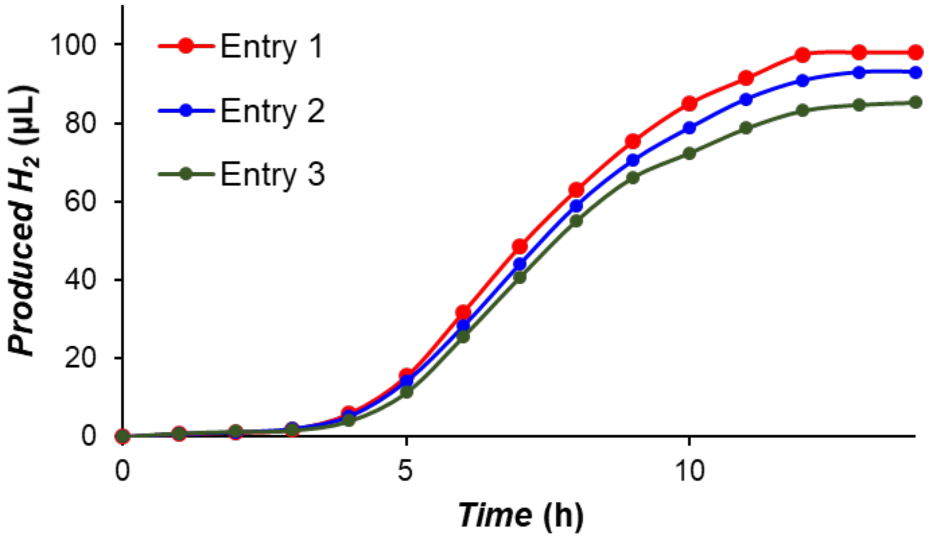

Figure S10. Time-courses of volume of hydrogen produced during photo-reaction (Table 1, Experiment 3).

Table S4. Time-courses of volume and mole of hydrogen produced during photo-reaction and the average values (Table 1, Experiment 5).

Time (h)	Entry 1		Entry 2		Entry 3		Average		
	vol (μL)	μmol	vol (μL)	μmol	vol (μL)	μmol	vol (μL)	μmol	standard deviation
0	0.00	0.00	0.00	0.00	0.00	0.00	0.00	0.00	0.00
1	1.10	0.05	0.54	0.02	0.47	0.02	0.70	0.03	0.35
2	2.40	0.11	0.91	0.04	1.85	0.08	1.72	0.08	0.75
3	8.90	0.40	4.89	0.22	10.20	0.46	8.00	0.36	2.77
4	23.65	1.06	17.05	0.76	27.26	1.22	22.65	1.01	5.18
5	41.69	1.86	36.54	1.63	43.62	1.95	40.62	1.81	3.66
6	58.71	2.62	55.16	2.46	59.96	2.68	57.94	2.59	2.49
7	72.63	3.24	70.95	3.17	69.17	3.09	70.92	3.17	1.73
8	79.95	3.57	79.11	3.53	73.64	3.29	77.57	3.46	3.43
9	82.06	3.66	83.92	3.75	75.98	3.39	80.65	3.60	4.15
10	83.13	3.71	84.99	3.79	77.83	3.47	81.98	3.66	3.72
12	83.13	3.71	84.99	3.79	77.83	3.47	81.98	3.66	3.72

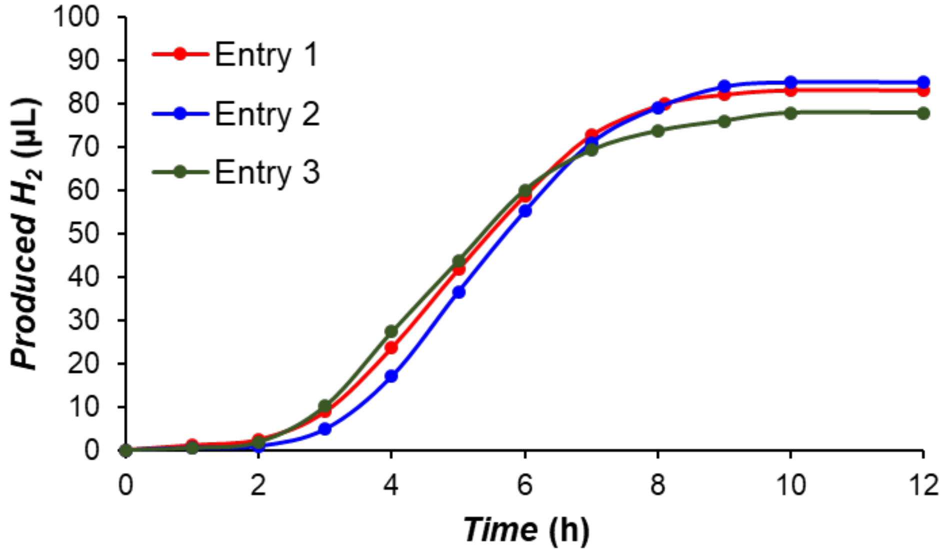

Figure S11. Time-courses of volume of hydrogen produced during photo-reaction (Table 1, Experiment 5).

Table S5 Time-courses of volume and mole of hydrogen produced during photo-reaction and the average values (Table 1, Experiment 6).

Time (h)	Entry 1		Entry 2		Entry 3		Average		
	vol (µL)	µmol	vol (µL)	µmol	vol (µL)	µmol	vol (µL)	µmol	standard deviation
0	0.00	0.00	0.00	0.00	0.00	0.00	0.00	0.00	0.00
1	1.00	0.04	1.23	0.06	0.71	0.03	0.98	0.04	0.26
2	2.14	0.10	2.29	0.10	0.82	0.04	1.75	0.08	0.81
3	2.59	0.12	3.80	0.17	1.46	0.07	2.61	0.12	1.17
4	3.58	0.16	8.82	0.39	5.47	0.24	5.96	0.27	2.65
5	8.76	0.39	22.92	1.02	16.44	0.73	16.04	0.72	7.09
6	21.68	0.97	43.62	1.95	35.90	1.60	33.73	1.51	11.13
7	40.00	1.79	62.65	2.80	57.70	2.58	53.45	2.39	11.91
8	58.86	2.63	80.57	3.60	73.56	3.28	71.00	3.17	11.08
9	74.77	3.34	94.22	4.21	89.01	3.97	86.00	3.84	10.07
10	89.16	3.98	105.66	4.72	104.45	4.66	99.76	4.45	9.19
11	101.90	4.55	111.13	4.96	112.34	5.02	108.46	4.84	5.71
12	111.70	4.99	116.13	5.18	116.13	5.18	114.65	5.12	2.56
13	115.45	5.15	116.39	5.20	116.13	5.18	115.99	5.18	0.49
14	120.26	5.37	116.39	5.20	116.13	5.18	117.59	5.25	2.31
15	121.64	5.43	116.39	5.20	116.13	5.18	118.05	5.27	3.11
16	121.64	5.43	116.39	5.20	116.13	5.18	118.05	5.27	3.11

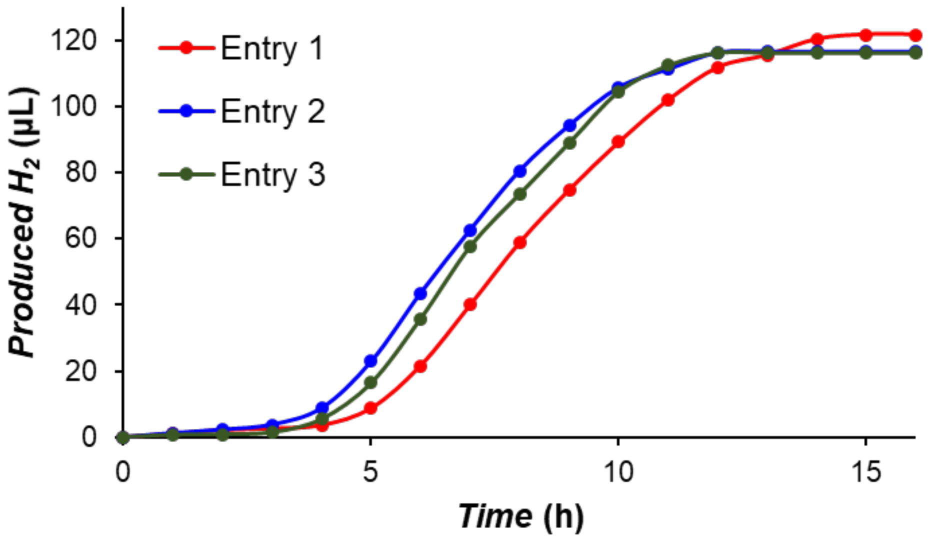

Figure S12. Time-courses of volume of hydrogen produced during photo-reaction (Table 1, Experiment 6).

References

[1] P. Wardman, *J. Phys. Chem. Ref. Data* **1989**, *18(4)*, 1637.

[2] Y. Pellegrin, ; F. Odobel, *Comptes Rendus Chim.* **2017**, *20(3)*, 283.
